# 3M™ Petrifilm™ Rapid Yeast and Mold Count Plate for the Enumeration of Yeasts and Molds in Dried Cannabis Flower: AOAC *Official Method*^SM^ 2014.05

**DOI:** 10.1093/jaoacint/qsac130

**Published:** 2022-10-29

**Authors:** April Schumacher, Cari Lingle, Karen M Silbernagel

**Affiliations:** 3M Center, 3M Food Safety Department, Bldg. 260-6B-01, St. Paul, MN 55144-1000, USA; 3M Center, 3M Food Safety Department, Bldg. 260-6B-01, St. Paul, MN 55144-1000, USA; 3M Center, 3M Food Safety Department, Bldg. 260-6B-01, St. Paul, MN 55144-1000, USA

## Abstract

**Background:**

The 3M^TM^ Petrifilm™ Rapid Yeast and Mold Count (RYM) Plate is a sample-ready culture medium system which contains nutrients supplemented with antibiotics, a cold-water-soluble gelling agent, and an indicator system that facilitates yeast and mold enumeration.

**Objective:**

The 3M Petrifilm RYM Plate was validated for the enumeration of yeast and mold in dried cannabis flower through the AOAC Emergency Response Validation process.

**Methods:**

The performance of the 3M Petrifilm RYM Plate was compared to dichloran rose bengal chloramphenicol (DRBC) agar. Matrix data were normalized by log_10_ transformation and performance indicators included repeatability, difference of means, and inclusivity/exclusivity.

**Results:**

These studies demonstrated the 3M Petrifilm RYM Plate method detects and enumerates yeasts and molds from dried cannabis flower at low, medium, and high contamination levels. The average log counts at 25 or 28°C for 60 to 72 h were equivalent to the average log counts of the DRBC reference method at low, medium and high levels. In strain studies, all 71 yeasts and molds tested produced typical colony morphology on 3M Petrifilm RYM Plates. Of the 32 non-target bacterial strains tested, none were detected on 3M Petrifilm RYM Plates.

**Conclusion:**

The 3M Petrifilm RYM Plate is a reliable method for the enumeration of live yeast and mold in dried cannabis flower.

**Highlights:**

The 3M Petrifilm RYM Plate allows for rapid detection of yeast and mold within 60 to 72 h of incubation. Up to 40 sample-ready plates can be stacked during incubation to save space.

Yeast and mold are widespread in nature, can be found in air, water, soil, and vegetation, and can grow in a wide range of environmental conditions. Cannabis plants are grown in both outdoor and indoor conditions. Plants grown outdoors are exposed to wider ranges and larger populations of fungal species; however, improper watering, type of soil and fertilizer, and poor air circulation can increase the chance of mold growth in indoor environments. Human handling during harvest increases the risk of secondary contamination for both indoor- and outdoor-grown cannabis. The final product could develop fungi or their growth by-products if humidity and temperature levels of drying and curing rooms are not carefully controlled. Total yeast and mold count (TYMC) is used as an indicator of the overall cleanliness of the product’s life cycle, from growing, processing, and handling to storage. Product with high TYMC can be detrimental to both consumers and cultivators.

While the majority of mold and yeast present in the environment are harmless, certain fungi cause spoilage and produce mycotoxins, a by-product that is toxic to humans and animals. Several yeast and mold have been found to be prevalent in cannabis, including *Cryptococcus, Mucor, Aspergillus fumigatus, A. niger,* and *A. flavus* (1, 2). *Aspergillus* species *niger, flavus,* and *fumigatus* are known for aflatoxin production, a type of dangerous mycotoxin that can be lethal (3). For this reason, regulations exist to limit the allowable TYMC counts for the purposes of protecting consumer safety (4).

The 3M^TM^ Petrifilm^TM^ Rapid Yeast and Mold Count (RYM) Plate is a sample-ready culture medium system which contains nutrients supplemented with antibiotics, a cold-water-soluble gelling agent, and an indicator system that facilitates yeast and mold enumeration.

The performance of the 3M Petrifilm RYM Plate method was previously shown to be comparable to ISO 21527:2008 parts 1 and 2, and the U.S. Food and Drug Administration *Bacteriological Analytical Manual* Ch. 18 (FDA-BAM Ch. 18) reference methods (5–7) for enumeration of yeast and mold in food and environmental surfaces at 25 ± 1°C and 28 ± 1°C after 48 to 60 h (high-water activity matrixes: yogurt, frozen bread dough, fermented salami, sour cream, ready-made pie, raw frozen ground beef patties (77% lean), ready-to-eat deli sandwiches, sliced apples; low-water activity matrixes: raw almonds, dehydrated soup; and environmental surfaces: stainless steel, sealed concrete, and rubber (8–10)). The method has Final Action status as *Official Methods of Analysis*^SM^ (OMA) **2014.05** (11). The current matrix extension study compares the performance of the 3M Petrifilm RYM to dichloran rose bengal chloramphenicol (DRBC) agar for the enumeration of yeast and mold in dried cannabis flower (9-tetrahydrocannabinol [THC] >0.3%) at 60 to 72 h.

## Matrix Extension Validation Study

This validation study was conducted as an Emergency Response Validation (ERV) process within the AOAC Research Institute (RI) *Performance Tested Method*^SM^ (PTM) program. The validation followed the AOAC INTERNATIONAL *Methods Committee Guidelines for Validation of Microbiological Methods for Food and Environmental Surfaces* (12) and *Standard Method Performance Requirements (SMPR*) for Viable Yeast and Mold Count Enumeration in Cannabis and Cannabis Products (13), which was developed by the AOAC Cannabis Analytical Science Program. Inclusivity and exclusivity testing was conducted by 3M (St. Paul, MN 55144–1000, USA), ADRIA Développement (ZA Creac’h Gwen F-29196 QUIMPER Cedex, France), and Q Laboratories (Cincinnati, OH, USA). Cannabis flower matrix study materials were prepared by Steadfast Analytical Laboratory (Hazel Park, MI, USA). Test portions were blind-coded and provided to North Coast Testing Laboratories of Michigan, (Adrian, MI, USA) for analysis using the 3M Petrifilm RYM Plate. Additional cannabis flower matrix study materials were prepared, blind-coded, and analyzed by Aurum Laboratories (Durango, CO, USA).

### Inclusivity/Exclusivity Study

An inclusivity/exclusivity study of the 3M Petrifilm RYM Plate was previously performed using 42 strains of yeast and mold and 4 strains of non-target organisms as part of the AOAC PTM 121301 and AOAC OMA **2014.05** validation studies (8, 9). Evaluation of target and non-target strains was not required for yeast and mold methods per AOAC Appendix J (11) when AOAC PTM 121301 and OMA **2014.05** studies were conducted. A select number of target and non-target organisms were included in the validation studies as supplemental data on the performance of the method. For the organisms obtained from Microbiologics, Inc. as LYFO DISK^®^ preparations, one pellet was added to 10 mL pre-warmed (37°C) 0.1% peptone water (PW) and then mixed on a vortex mixer until the pellet was completely dissolved. The pellet suspension was serially diluted as needed (the pellet counts provided by the manufacturer served as guidance in establishing the dilution scheme). The 3M Petrifilm RYM Plates were then incubated at 25 and 28°C and enumerated at 48 and 60 h.

The ERV protocol required data for specific inclusivity and exclusivity organisms commonly found in cannabis matrixes. Additional strains to satisfy this requirement were provided using a subset of data from ADRIA Développement for a previously conducted NF Validation by AFNOR certification (3M 01/13-07/14) in comparison to ISO 21527 part 1 and part 2 (14 inclusivity, 20 exclusivity), or were tested by Q Laboratories (17 inclusivity, 8 exclusivity).

In the study conducted by ADRIA Développement, the inclusivity strains were grown in Sabouraud broth at 25°C. The non-target strains were grown in appropriate media at appropriate temperatures. All organisms were tested at two incubation temperatures, 25 ± 1°C and 28 ± 1°C. Plates were examined and results recorded at 48 h (data not published in NF Validation by AFNOR certification) and 60 and 72 h (10).

In the study conducted by Q Laboratories, yeast organisms were propagated from a stock culture stored at −70°C to potato dextrose broth and incubated at temperatures optimal for growth. Following incubation, yeast organisms were diluted to 100× the LOD of the 3M Petrifilm RYM Plates. Mold organisms were propagated from a stock culture stored at −70°C to Sabouraud dextrose agar and incubated for 5–7 days at 30 ± 1°C. Following incubation, mold spores were harvested for inclusivity testing by washing cultures with Butterfield’s phosphate-buffered dilution water. The mold wash was then diluted to 100× the LOD of the 3M Petrifilm RYM Plates.

Exclusivity organisms were propagated from a stock culture stored at −70°C to trypticase soy agar with 5% sheep blood and incubated at conditions optimal for growth. Following incubation exclusivity organisms were transferred to the non-selective brain heart infusion broth and incubated at conditions optimal for growth. Exclusivity cultures were analyzed undiluted.

All organisms were randomized in a blind-coded study and plated onto 3M Petrifilm RYM Plates as indicated in the instructions for use. All organisms were tested at two incubation temperatures, 25 ± 1°C and 28 ± 1°C. Plates were examined and results recorded at 48 and 60 h Colonies were determined to be positive or negative based on the product instructions.

### Matrix Study

Cannabis test materials were prepared by Steadfast Analytical from an inventory of retained samples from its Michigan-licensed grower, patient, and caregiver customers. Steadfast combined cannabis samples to produce batch materials targeting at least 1000 g at a low level [<1000 colony-forming units (cfu)/g), a medium level (1000–10 000 cfu/g), and a high level (10 000–100 000 cfu/g). Batches were manually mixed in an aseptic manner until homogeneous. For each contamination level, five replicate test portions (10 g) were quantified by spread plating aliquots of diluted test portions onto DRBC agar plates. Table 1 summarizes the average cfu/g of yeast and mold for each contamination level that was provided to laboratories for analysis in the study.

**Table 1.** Average contamination level of yeast and mold in test batches

Batch	*n* ^a^	DRBC^b^(cfu^c^/g)
Low	5	350
Medium	5	5600
High	5	48 000

a *n* = Number of replicates.

b DRBC = dichloran rose bengal chloramphenicol agar.

c cfu/g = Colony-forming units per gram of cannabis material tested.

Individual 10 g test portions from each contamination level were placed in sterile filter Whirl-Pak bags. Five bagged test portions from each of the three contamination levels were selected for each candidate method participating in the ERV project. Test portions were assigned an identification tag in Michigan’s Marijuana Regulatory Agency seed-to-sale system for distribution and tracking. This served to blind-code the contamination level of the test portions. The test portions were also assigned random sample numbers for reporting results to AOAC.

Personnel from each of the participating independent laboratories were responsible for picking up and transporting the test portions to their laboratories on Monday, December 7, 2020. Participating laboratories were instructed to analyze samples on Tuesday, December 8, 2020, following the user guides provided with the candidate methods. In addition to the candidate methods, all test portions were enumerated using DRBC agar as described in the DRBC reference section. For the 3M Petrifilm RYM Plate method, North Coast Testing Laboratories of Michigan conducted the matrix evaluation.

A second set of cannabis samples targeting a low level (approximately 1000 cfu/g) and a high level (approximately 100 000 cfu/g) were prepared by Aurum Laboratories. A 50–60 g sample of cannabis flower matrix was made by combining previously tested sample inventory. Cannabis flower samples that previously tested at/or slightly above 1000 cfu/g were combined into one large sample. Cannabis flower samples that previously tested >74 000 cfu/g were combined into one large sample. The combined samples were homogenized using a sterile stainless steel mortar and pestle. A portion of the homogenized sample was used for cannabinoid analysis to confirm THC >0.3%. From each homogenized sample, five 10 g samples were aseptically weighed out into sterile Whirl-Pak filter bags. Samples were blind-coded prior to plating at Aurum Laboratories.

### Candidate Method

All analyses at both laboratories were performed using paired test portions. Test portions were prepared for analysis as described in the 3M Petrifilm RYM Plate method. Ten gram portions were homogenized in 90 mL 0.1% PW. Tenfold dilutions were made by transferring 10 mL into 90 mL PW and shaking 25 times in a 1-foot arc within 7 s to ensure homogeneity. A 1 mL aliquot from each dilution (10^−1^, 10^−2^, 10^−3^, and 10^−4^) was plated in duplicate on Petrifilm RYM Plates. Two sets of plates were prepared, one set incubated at 25 ± 1°C and the second set incubated at 28 ± 1°C. Plates were read and colonies recorded at 72 h (North Coast Testing Laboratories) or 60 h and 72 h (Aurum Laboratories). Plates containing counts between 10–150 were used to determine the final results. If mainly yeast are present, plates with 150 colonies are usually countable. When substantial amounts of mold were present and a more accurate count was obtained on the next dilution, the upper countable limit was lowered per the guidance in the 3M Petrifilm RYM Plate method and FDA-BAM Ch. 18. The counts from duplicate plates were averaged and used for the statistical analysis.

### Reference Method

Paired test portions, prepared following the candidate method dilution protocol, were confirmed by spread plating aliquots of each dilution onto DRBC agar plates. From the initial dilution of sample (10 g homogenized in 90 mL PW), 1.0 mL was spread plated across two DRBC agar plates (0.5 mL on each plate) in triplicate (six total DRBC agar plates) or across three DRBC agar plates (0.33 mL on each plate) in triplicate (nine total DRBC agar plates) at North Coast Laboratory or Aurum Laboratories, respectively. Additionally, 0.1 mL of the 10^−1^, 10^−2^, and 10^−3^ dilutions was plated in triplicate on DRBC to obtain the 10^−2^, 10^−3^, and 10^−4^ dilutions respectively. The agar plates were allowed to dry and were then incubated at 25 ± 1°C for 5–7 days before enumeration. Mold appeared as flat or fuzzy, spreading colonies with the natural pigmentation of the sporing structures, and yeast appeared as pink, smooth, raised colonies on DRBC agar plates (5). Plates containing counts between 10–150 colonies were enumerated as described in the FDA-BAM Ch. 18. The counts from triplicate plates were averaged and used for the statistical analysis.

**AOAC *Official Method* 2014.05**

**Enumeration of Yeast and Mold in Foods, Selected Surfaces,and Dried Cannabis Flower**

**3M Petrifilm™ Rapid Yeast and Mold Count Plate**

**First Action 2014**

**Final Action 2017**

**Revised First Action 2022 (for Cannabis Flower, THC >0.3%, Only)**

[Applicable to the enumeration of yeast and mold in the following high-water activity matrixes: yogurt, frozen bread dough, fermented salami, sour cream, ready-made pie, raw frozen ground beef patties (77% lean), ready-to-eat deli sandwiches, sliced apples; the following low-water activity matrixes: raw almonds, dehydrated soup, dried cannabis flower (THC > 0.3%); and the following environmental surfaces: stainless steel, sealed concrete, and rubber.]

*Caution*: After use, the diluents and 3M Petrifilm RYM Plates may contain microorganisms that may be a potential biohazard as several foodborne molds have the ability to produce toxic metabolites known as mycotoxins. If further identification of a mold species is required, appropriate personal protective equipment (PPE) should be used when top film is retracted and exposure to spores or mycotoxins may occur. When testing is complete, follow current industry standards for the disposal of contaminated waste. Consult the material safety data sheet for additional information and local regulations for disposal. For information on potential biohazards, reference *Biosafety in Microbiological and Biomedical Laboratories*, 6th Ed., Section VIII-B: Fungal Agents.

The 3M Petrifilm RYM Plates contain chloramphenicol and chlortetracycline, potent broad spectrum antibiotic drugs commonly used in yeast and mold enumeration. The drugs, when used in humans, is associated with many toxic effects. Care should be taken to avoid coming into direct contact with the gel on the plates.

*See* Tables **2014.05A** and **2014.05B** for a summary of results of the collaborative study. The result for each collaborating laboratory’s aerobic plate count analysis for each matrix is shown in Table **2014.05C**.

*See* Tables 2–9 for detailed results of the collaborative study [*J. AOAC Int.* **98**, 767(2015)].

### A. Principle

The 3M Petrifilm RYM Plate is a sample-ready culture medium system, which contains nutrients supplemented with antibiotics, a cold-water-soluble gelling agent, and an indicator system that facilitates yeast and mold enumeration. 3M Petrifilm RYM Plates are used for the enumeration of yeast and mold in as little as 48 h in the food and beverage industries and 60 to 72 h in the cannabis industry. 3M Food Safety is certified to International Organization for Standardization (ISO) 9001 for design and manufacturing.

### B. Apparatus and Reagents

- *3M Petrifilm Rapid Yeast and Mold Count Plate.*—Available from 3M Food Safety, St. Paul, MN, USA—Cat. No. 6475/6477.
- *Sterile diluents.—*0.1% peptone water (PW) or Butterfield’s phosphate buffered dilution water (BPBD).
- *Pipets.—*Capable of 1000 µL or a serological pipet.
- *Sterile pipet tips.—*Capable of 1000 µL.
- *Stomacher.—*Seward or equivalent.
- *Filter stomacher bags.*—Seward or equivalent.
- *3M Petrifilm Flat Spreader.—Cat. No. 6425*
- *3M Swab Sampler with 10 mL Letheen broth.—*Cat. No. RS96010LET or equivalent.
- *Incubators.—*Capable of maintaining 25 ± 1°C and 28 ± 1°C and having a solid front to maintain a dark interior.
- *Refrigerator.—*Capable of maintaining 2–8°C, for storing the 3M Petrifilm RYM Plates.
- *Standard colony counter or illuminated magnifier.*

### C. General Instructions

- Store unopened 3M Petrifilm RYM Plate pouches refrigerated or frozen (–20 to 8°C/–4 to 46 °F). Just prior to use, allow unopened pouches to come to room temperature before opening (20–25°C/<60% RH). Return unused 3M Petrifilm RYM Plates to the pouch. Seal by folding the end of the pouch over and applying adhesive tape. To prevent exposure to moisture, do not refrigerate opened pouches. Store resealed pouches in a cool dry place (20–25°C/<60% RH) for no longer than 4 weeks. It is recommended that resealed pouches of 3M Petrifilm RYM Plates be stored in a freezer if the laboratory temperature exceeds 25°C (77 °F) and/or the laboratory is located in a region where the relative humidity (RH) exceeds 60% (with the exception of air-conditioned premises).
  To store opened pouches in a freezer, place 3M Petrifilm RYM Plates in a sealable container.
  Post-incubation 3M Petrifilm RYM Plates can be stored at –10 to –20°C for up to 7 days.
- Follow all instructions carefully. Failure to do so may lead to inaccurate results.

### D. Sample Preparation

- Aseptically prepare a 1:10 dilution of each test portion.
  1. *Dairy products*.—Pipet 11 mL or weigh 11 g sample into 99 mL sterile 0.1% PW. Shake 25 times to homogenize.
  2. *All other foods.—*Weigh out 25 g sample from test portion into a sterile stomacher bag and dilute with 225 mL 0.1% PW; stomach at high speed to homogenize.
  3. *Dried cannabis flower (THC >0.3%).—*Weigh out 10 g sample from test portion into a sterile stomacher bag and dilute with 90 mL sterile 0.1% PW. Shake 25 times to homogenize.
  4. *Environmental surfaces*.*—*Mix or shake swab vigorously in Letheen broth.
- Prepare 10-fold serial dilutions in 0.1% PW or BPBD. Environmental surface samples may be plated directly as needed.
- Place a 3M Petrifilm RYM Plate on a flat, level surface for each dilution to be tested.
- Lift the top of the film. Dispense 1 mL of each dilution onto the center of the bottom film of each plate.
- Roll the film down onto the sample.
- Place the 3M Petrifilm Flat Spreader on the center of the plate. Press gently on the center of the spreader to distribute the sample evenly. Spread the inoculum over the entire 3M Petrifilm RYM Plate growth area before the gel is formed. *Do not slide the spreader across the film.*
- Remove the spreader and leave the plate undisturbed for at least 1 min to permit the gel to form.
- Incubate the 3M Petrifilm RYM Plates at 25 or 28°C in a horizontal position with the clear side up in stacks of no more than 40.
  1. *For food or environmental samples*:*—*Enumerate plates after 48 h of incubation. If colonies appear faint, allow for an additional 12 h of incubation time for enhanced interpretation.
  2. *For dried cannabis flower*:*—*Enumerate plates at 60 to 72 h of incubation.
- 3M Petrifilm RYM Plates can be counted using a standard colony counter with the use of a back light or an illuminated magnifier to assist with the estimated enumeration. Do not count colonies on the foam dam since they are removed from the nutrient medium.
- Yeast colonies appear raised and small with defined edges. Colonies may appear pink/tan or blue/green in color.
- Mold colonies appear flat with a dark center and diffused edges. Colonies may appear blue/green to variable upon prolonged incubation. See Table **2014.05D** for yeast and mold appearance.
- The circular growth area is approximately 30 cm^2^. Plates containing greater than 150 colonies can be either estimated or recorded as too numerous to count (TNTC). Estimation can only be done by counting the number of colonies in one or more representative squares and determining the average number per square. The average number can be multiplied by 30 to determine the estimated count per plate. If a more accurate count is required, the sample will need to be retested at higher dilutions. When the sample contains substantial amounts of mold, depending on the type of mold, the upper countable limit may be at user discretion.
- Samples may occasionally show interference on the 3M Petrifilm RYM Plates, for example:
  1. Uniform blue background color (often seen from the organisms used in cultured products). These should not be counted as TNTC.
  2. Intense pinpoint blue specks (often seen with spices, granulated products, or dried cannabis flower).
- Report final results as cfu/g.
- If required, colonies may be isolated for further identification by direct microscopy or biochemical analysis. Lift the top film and pick the colony from the gel.

### Results

- *Inclusivity/Exclusivity*.—Results from the inclusivity and exclusivity testing conducted at Q Laboratories have been combined with the results from ADRIA Développement and the original 3M Petrifilm RYM Plate AOAC validation and are presented in Table 2 (inclusivity) and Table 3 (exclusivity). Seventy-one yeasts and molds tested showed typical colony morphology and thus were considered “positive” on 3M Petrifilm RYM Plates. Sixty-three out of 71 strains grew at both temperatures, 25 ± 1°C and 28 ± 1°C, and at both time points, 48 and 60 h. Two strains were not visible at 25 ± 1°C at 48 h but showed growth at 25 ± 1°C at 60 h and at 48 and 60 h at 28 ± 1°C. Five strains were not visible at 48 h at 25 ± 1°C or 28 ± 1°C but showed growth at 60 h at both temperatures. One strain was not visible at 48 h at 25 ± 1°C or 28 ± 1°C, but showed growth at 72 h at 25 ± 1°C and 60 h at 28 ± 1°C. None of the exclusivity strains were detected on the 3M Petrifilm RYM Plates at either temperature or incubation time.
- *Matrix study.*—Statistical analysis was conducted for each contamination level comparing the candidate method result to cfu/g obtained on the DRBC agar plates. For each test portion, results were logarithmically (log_10_) transformed using the equation cfu/g + 0.1, according to the Least Cost Formulations, Ltd (2020) *Paired Method Analysis for Micro Testing* Version 1.2 (Virginia Beach, VA, USA). After transformation, replicate test portion results for each contamination level for each method were averaged, and the difference of means between methods with 90 and 95% confidence intervals were determined. Repeatability and RSD of repeatability were also calculated. The matrix study data are presented in Tables 4–7.

**Table 2.** Inclusivity results for the 3M Petrifilm RYM Plates

No.	Organism	Source	Origin	25°C 48 h Results	25°C 60 h Results	28°C 48 h Results	28°C 60 h Results
1	*Alternaria alternata* ^a^	ATCC^b^ 66981	*Arachis hypogaea*	+^c^	+	+	+
2	*Arthrinium* species *(aureum)*^a^	ATCC 56042	Not available	+	+	+	+
3	*Aspergillus aculeatus*	ATCC 56925	Grape	+	+	+	+
4	*Aspergillus brasiliensis* ^a^ ^,^ ^d^	ATCC 16404	Blueberry	+	+	+	+
5	*Aspergillus caesiellus* ^a^	ATCC 42693	Dried chilies	+	+	+	+
6	*Aspergillus carbonarius*	ATCC 6276	Not available	+	+	+	+
7	*Aspergillus flavus*	ATCC 9643	Shoe sole	+	+	+	+
8	*Aspergillus fumigatus* ^f^	ATCC 204305	Human sputum	−^c^	+	+	+
9	*Aspergillus japonicus*	ATCC 52036	Soil	+	+	+	+
10	*Aspergillus niger* ^a^	ATCC 6275	Not available	+	+	+	+
11	*Aspergillus niger* (re*-*classified as *brasiliensis*)	3M^j^ M6	Not available	+	+	+	+
12	*Aspergillus oryzae*	ATCC 10124	Not available	+	+	+	+
13	*Aspergillus terreus* ^a^	ATCC 1012	Soil	+	+	+	+
14	*Aspergillus ustus* (re-classified as *Aspergillus puniceus*)	ATCC 10760	Greenhouse soil	+	+	+	+
15	*Aureobasidium* species (pullulans)^a^	ATCC 15233	Painted wood	+	+	+	+
16	*Botrytis species* ^f^	3M M97	Not available	–	+	+	+
17	*Candida albicans*	ATCC 10231	Not available	+	+	+	+
18	*Candida catenulata* (re-classified as *Diutina catenulata*)	ATCC 10565	Human feces	+	+	+	+
19	*Candida glabralta*	ATCC 15126	Not Available	+	+	+	+
20	*Candida guillermondii* (re-classified as *Meyerozyma guilliermodii*)	ATCC 6260	Not available	+	+	+	+
21	*Candida kefyr*	ATCC 204093	Not available	+	+	+	+
22	*Candida krusei*	ATCC 14243	Not available	+	+	+	+
23	*Candida lusitaniae*	ATCC 34449	Pig	+	+	+	+
24	*Candida sphaerica*	Microbiologics^e^	Not available	+	+	+	+
25	*Candida tropicalis*	ATCC 13803	Not available	+	+	+	+
26	*Chaetomium globosum* ^a^ ^,^ ^d^	ATCC 6205	Stored cotton	+	+	+	+
27	*Cladosporium cladosporioides* ^g^	Ad1405^h^	Bakery	+	+	+	+
28	*Cladosporium herbarum* (re-classified as *Cladosporium macrocarpum*)	ATCC 76226	Sugar beet leaf	+	+	+	+
29	*Cladosporium* spp. *cladosporoides*	ATCC 16022	Painted floor	+	+	+	+
30	*Cryptococcus liquefaciens* ^g^	Adria^h^ 1041	Environment	+	+	+	+
31	*Cryptococcus magnus* ^g^	Adria 1040	Environment	+	+	+	+
32	*Curvularia lunata* ^a^	ATCC 12017	Not available	+	+	+	+
33	*Trichosporon mucoides* (re-classified as *Cutaneotrichosporon dermatis*)	ATCC 201382	Not available	+	+	+	+
34	*Debaryomyces hansenii*	Microbiologics	Cheese and milk	+	+	+	+
35	*Fusarium oxysporum* ^a^	QL^i^ 0567126A	Environmental isolate	+	+	+	+
36	*Fusarium proliferatum* ^a^	QL 0567112.1C	Environmental isolate	+	+	+	+
37	*Fusarium solani* ^a^	QL 345317.4B	Environmental isolate	+	+	+	+
38	*Geotrichum candidum* ^g^	ATCC 204307	Dairy	+	+	+	+
39	*Geotrichum candidum* (re-classified as *Galactomyces candidus*)	ATCC 34614	Clotted carrot	+	+	+	+
40	*Geotrichum capitatum*	Microbiologics 0482 L	Not available	+	+	+	+
41	*Hanseniaspora uvarum* ^g^	CLIB^h^ 303	Oenology	+	+	+	+
42	*Hansenuela anomala*	3M Y28	Not availbale	+	+	+	+
43	*Kluyveromyces lactis*	ATCC 8563	Creamery	+	+	+	+
44	*Kluyveromyces lactis*	ATCC 10689	Cheese	+	+	+	+
45	*Kluyveromyces marxianus* ^g^	CLIB 720	Dairy	+	+	+	+
46	*Mucor plumbeus* ^g^	Adria M10	Food	+	+	+	+
47	*Mucor racemosus*	NCPF 7650	Not available	+	+	+	+
48	*Paecilomyces* sp. (M10)	Microbiologics 0287E4	Not available	+	+	+	+
49	*Paecilomyces variotii* ^g^	Adria M6	Food	–	+72 hr^k^	–	+
50	*Penicillium aurantiogriseum* (re-classified as *Penicillium venetum*)	ATCC 16025	*Hyacinthus* sp. bulb	–	+	–	+
51	*Penicillium chrysogenum*	ATCC 10106	Cheese	+	+	+	+
52	*Penicillium citreonigrum* ^g^	Adria 1052	Environment	+	+	+	+
53	*Penicillium roqueforti* ^g^	Adria M1	Dairy	–	+	–	+
54	*Phoma glomerata* ^g^	Adria M4	Food	+	+	+	+
55	*Phytophthora infestans* ^a^	ATCC MYA 1113	Potato tuber	+	+	+	+
56	*Purpureocillium* species (*lilacinum*)^a^	ATCC 10114	Soil	+	+	+	+
57	*Rhizopus oryzae* ^a^	ATCC 9363	Soy sauce	+	+	+	+
58	*Rhizopus stolonifera* ^a^	QL 14181-2A	Not available	+	+	+	+
59	*Rhodotorula mucaliginosa*	ATCC 66034	Not available	+	+	+	+
60	*Rhodotorula graminis* ^g^	Adria 1032	Environment	+	+	+	+
61	*Saccharomyces cerevisiae*	ATCC 7754	Not available	+	+	+	+
62	*Saccharomyces lactis*	Microbiologics	Not available	+	+	+	+
63	*Scopulariopsis acremonium*	ATCC 58636	Chicken house soil	+	+	+	+
64	*Talaromyces pinophilus (Penicillium pinophilum)* ^a^	NRRL 11797	Corn	+	+	+	+
65	*Trichoderma virens*	ATCC 9645	Not available	+	+	+	+
66	*Trichoderma virride* ^g^	Adria M2	Food	–	+	–	+
67	*Ustilago* spp.	Microbiologics	Not available	+	+	+	+
68	*Yarrowia lipolytica* ^g^	CLIB 183	Food	+	+	+	+
69	*Yarrowia lipolytica*	3M Culture collection^j^	Cheese	+	+	+	+
70	*Zygosaccharomyces bailii* (re-classified as *Zygosaccharomyces parabailii*)	ATCC MYA-4549	Salad dressing	–	+	–	+
71	*Zygosaccharomyces rouxii*	ATCC 28253	Processed prunes	–	+	–	+

a Strain tested by Q Laboratories to satisfy AOAC SMPR 2021.009 requirements (13). Strains without footnote “a” or “g” were tested as part of AOAC PTM 121301 and AOAC OMA **2014.05** (8, 11)

b ATCC = American Type Culture Collection, Manassas, VA, USA.

c The “+” symbol indicates typical colony morphology observed on 3M Petrifilm RYM Plates and the “-” symbol indicates no growth observed on 3M Petrifilm RYM Plates.

d Strain was tested by Q Laboratories and was also part of the of AOAC PTM 121301 and AOAC OMA **2014.05** (8, 11).

e Strain purchased from Microbiologics, source detail was taken from AOAC PTM 121301 (8).

f Prefer a higher temperature for growth. These molds are visible on the RYM Plates within 48 h at 28°C but require 60 h to grow at 25°C.

g Strain tested by ADRIA Développement for NF Validation by AFNOR certification in comparison to ISO 21527 (5, 6). Strains added to satisfy AOAC SMPR 2021.009 requirements (13). Strains without footnote “a” or “g” were tested as part of AOAC PTM 121301 and AOAC OMA **2014.05** (8, 11).

h Adria = ADRIA Développement Culture Collection, ZA Creac’h Gwen F-29196 QUIMPER Cedex, France.

i QL = Q Laboratories Culture Collection, Cincinnati, OH, USA.

j 3M = 3M Culture Collection, St. Paul, MN, USA, source detail was taken from AOAC PTM 121301 (8).

k *Paecilomyces variotii* were visible on the RYM Plates at 72 h at 25 °C.

**Table 3.** Exclusivity results for the 3M Petrifilm RYM Plates

No.	Organism	Source	Origin	25°C 48 h Results	25°C 60 h Results	28°C 48 h Results	28°C 60 h Results
1	*Aeromonas hydrophila*	ATCC^a^ 7965	Not available	−^c^	–	–	–
2	*Bacillus subtilis*	ATCC 6633	Not available	–	–	–	–
3	*Bacillus subtilis* ^b^	Adria^d^ 863	Not available	–	–	–	–
4	*Bacillus weihenstephanesis* ^b^	Adria 778	Not available	–	–	–	–
5	*Brocotix thermospacta* ^b^	EN^d^ 15/29	Not available	–	–	–	–
6	*Bruttiaux agrestis* ^b^	Adria 117	Not available	–	–	–	–
7	*Carnobacterium piscicola* ^b^	Adria 369	Not available	–	–	–	–
8	*Citrobacter braakii* ^e^	ATCC 43162	Clinical isolate	–	–	–	–
9	*Corynebacterium* spp.^b^	Adria 361	Not available	–	–	–	–
10	*Edwardsiella tarda* ^e^	ATCC 15947	Human feces	–	–	–	–
11	*Enterobacter cloacae* ^e^	ATCC 13047	Spinal fluid	–	–	–	–
12	*Enterococcus faecalis*	ATCC 14506	Not available	–	–	–	–
13	*Enterococcus faecalis* ^b^	Adria 288	Not available	–	–	–	–
14	*Erwinia amylovora* ^e^	ATCC 51852	Plant	–	–	–	–
15	*Escherichia coli*	ATCC 25922	Clinical isolate	–	–	–	–
16	*Escherichia coli* O157: H7^e^	ATCC 43895	Raw hamburger	–	–	–	–
17	*Hafnia alvei* ^e^	ATCC 51815	Milk	–	–	–	–
18	*Klebsiella oxytoca* ^e^	ATCC 43165	Clinical isolate	–	–	–	–
19	*Lactobacilus plantarum* ^b^	Adria 70	Not available	–	–	–	–
20	*Leclecia adecarboxylata* ^b^	Adria 707	Not available	–	–	–	–
21	*Leuconostoc oenos* ^b^	Adria 73	Not available	–	–	–	–
22	*Listeria innocua* ^b^	Adria 644	Not available	–	–	–	–
23	*Micrococcus leteus* ^b^	Adria 438	Not available	–	–	–	–
24	*Moraxella* ^b^	Adria 51.11	Not available	–	–	–	–
25	*Plesiomonas shigelloides* ^b^	Adria 673	Not available	–	–	–	–
26	*Pseudomonas fluorescens* ^b^	Adria 16	Not available	–	–	–	–
27	*Pseudomonas putida* ^b^	Adria 4	Not available	–	–	–	–
28	*Ralstonia pickettii* ^e^	ATCC 27511	Clinical isolate	–	–	–	–
29	*Rhanella aqatilis* ^b^	Adria 67	Not available	–	–	–	–
30	*shewanella putrefaceins* ^b^	EN 15/34	Not available	–	–	–	–
31	*Staphylococcus aureus* ^b^	Adria 904	Not available	–	–	–	–
32	*Staphylococcus epidermidis* ^b^	Adria 931	Not available	–	–	–	–

a ATCC = American Type Culture Collection, Manassas, VA, USA.

b Strain tested by ADRIA Développement for NF Validation by AFNOR certification in comparison to ISO 21527 (5, 6). Strains without a footnote were tested as part of AOAC PTM 121301 and AOAC OMA **2014.05** (8, 11)

c – = No growth observed on 3M Petrifilm RYM Plates.

d Adria = ADRIA Développement Culture Collection, ZA Creac’h Gwen F-29196 QUIMPER Cedex, France.

e Strain tested by Q Laboratories to satisfy AOAC SMPR 2021.009 requirements (13). Strains without a footnote were tested as part of AOAC PTM 121301 and AOAC OMA **2014.05** (8, 11).

**Table 4.** Matrix study: 3M Petrifilm RYM Plate 25°C at 72 h versus DRBC—difference of means

Matrix	Contamination level	3M Petrifilm RYM Plate, 25°C; 72 h		DRBC		DOM^d^	SE^e^	90% CI^a^		95% CI	
		Mean^b^	s_r_^c^	Mean	s_r_			LCL^f^	UCL^g^	LCL	UCL
Cannabis Flower^h^	Low	3.281	0.094	3.688	0.218	−0.407	0.106	−0.577	−0.236	−0.629	−0.185
	Med	3.816	0.042	3.906	0.047	−0.09	0.028	−0.12	−0.06	−0.129	−0.051
	High	5.139	0.063	5.145	0.204	−0.005	0.064	0.143	0.132	−0.184	0.174
Cannabis Flower^i^	Low	2.850	0.222	3.151	0.211	−0.301	0.121	−0.468	0.047	−0.403	−0.199
	High	5.435	0.180	5.539	0.143	−0.105	0.075	−0.264	0.055	−0.379	−0.222

a CI = Confidence interval for DOM.

b Mean of five replicate portions, after logarithmic transformation: log10[cfu/g + (0.1)f] where f is the smallest reportable result.

c Repeatability standard deviation.

d DOM = Difference of means.

e Standard error of the mean difference for paired analysis.

f LCL = Lower confidence limit for DOM.

g UCL = Upper confidence limit for DOM.

h Matrix samples tested at North Coast Laboratories.

i Matrix samples tested at Aurum Laboratories.

**Table 5.** Matrix study: 3M Petrifilm RYM Plate 25°C at 60 h versus DRBC—difference of means

Matrix	Contamination level	3M Petrifilm RYM Plate, 25°C; 60 h		DRBC		DOM^d^	SE^e^	90% CI^a^		95% CI	
		Mean^b^	s_r_^c^	Mean	s_r_			LCL^f^	UCL^g^	LCL	UCL
Cannabis Flower^h^	Low	2.788	0.217	3.151	0.211	−0.363	0.113	−0.427	−0.299	−0.446	−0.280
	High	5.392	0.166	5.539	0.143	−0.147	0.072	−0.302	0.007	−0.348	0.054

a CI = Confidence interval for DOM.

b Mean of five replicate portions, after logarithmic transformation: log10[cfu/g + (0.1)f] where f is the smallest reportable result.

c Repeatability standard deviation.

d DOM = Difference of means.

e Standard error of the mean difference for paired analysis.

f LCL = Lower confidence limit for DOM.

g UCL = Upper confidence limit for DOM.

h Matrix samples tested at Aurum Laboratories.

**Table 6.** Matrix study: 3M Petrifilm RYM Plate 28°C at 72 h versus DRBC—difference of means

Matrix	Contamination level	3M Petrifilm RYM Plate, 28°C; 72 h		DRBC		DOM^d^	SE^e^	90% CI^a^		95% CI	
		Mean^b^	s_r_^c^	Mean	s_r_			LCL^f^	UCL^g^	LCL	UCL
Cannabis Flower^h^	Low	3.181	0.067	3.688	0.218	−0.507	0.082	−0.682	−0.332	−0.736	−0.279
	Med	3.697	0.039	3.906	0.047	−0.209	0.035	−0.284	−0.135	−0.306	−0.112
	High	5.065	0.208	5.145	0.204	−0.08	0.130	−0.172	0.013	−0.200	0.041
Cannabis Flower^i^	Low	2.838	0.214	3.061	0.226	−0.222	0.127	−0.493	0.048	−0.575	0.130
	High	5.450	0.176	5.539	0.143	−0.089	0.049	−0.194	0.016	−0.225	0.047

a CI = Confidence interval for DOM.

b Mean of five replicate portions, after logarithmic transformation: log10[cfu/g + (0.1)f] where f is the smallest reportable result.

c Repeatability standard deviation.

d DOM = Difference of means.

e Standard error of the mean difference for paired analysis.

f LCL = Lower confidence limit for DOM.

g UCL = Upper confidence limit for DOM.

h Matrix samples tested at North Coast Laboratories.

i Matrix samples tested at Aurum Laboratories.

**Table 7.** Matrix study: 3M Petrifilm RYM Plate 28°C at 60 h versus DRBC—difference of means

Matrix	Contamination level	3M Petrifilm RYM Plate, 28°C; 60 h		DRBC		DOM^d^	SE^e^	90% CI^a^		95% CI	
		Mean^b^	s_r_^c^	Mean	s_r_			LCL^f^	UCL^g^	LCL	UCL
Cannabis Flower^h^	Low	2.817	0.204	3.061	0.226	−0.244	0.114	−0.486	−0.001	−0.560	0.072
	High	5.432	0.188	5.539	0.143	−0.107	0.054	−0.221	0.007	−0.256	0.042

a CI = Confidence interval for DOM.

b Mean of five replicate portions, after logarithmic transformation: log10[cfu/g + (0.1)f] where f is the smallest reportable result.

c Repeatability standard deviation.

d DOM = Difference of means.

e Standard error of the mean difference for paired analysis.

f LCL = Lower confidence limit for DOM.

g UCL = Upper confidence limit for DOM.

h Matrix samples tested at Aurum Laboratories.

**Table 2014.05A.** Interlaboratory study results of 3M Petrifilm RYM versus FDA-BAM and ISO 21527 methods for frozen raw ground beef patties

		3M Petrifilm RYM method				BAM/ISO 21527 methods^a^						
Matrix	Lot	*n* ^b^	Mean^c^	s_r_	s_R_	*n*	Mean	s_r_	s_R_	*P*-value^d^	Difference of means	Reverse transformed mean difference^e^
Frozen raw ground beef patties												
25°C, 48 h	Control	11(0)	<1.00	—^f^	—	11(0)	<1.00	—	—	—	—	—
	Low	11(0)	2.12	0.41	0.41	11(1)	2.07	0.36	0.38	0.5323	0.05	14.34
	Medium	11(0)	3.52	0.10	0.10	11(0)	3.47	0.09	0.11	0.1637	0.05	360.10
	High	11(0)	4.65	0.13	0.14	11(0)	4.59	0.10	0.14	0.2266	0.06	5763.84
25°C, 60 h	Control	11(0)	<1.00	—	—	11(0)	<1.00	—	—	—	—	—
	Low	11(0)	2.14	0.36^g^	0.37	11(1)	2.07	0.36	0.38	0.3773	0.07	20.55
	Medium	11(0)	3.52	0.10	0.10	11(0)	3.47	0.09	0.11	0.1573	0.05	360.10
	High	11(0)	4.65	0.14	0.15	11(0)	4.59	0.10	0.14	0.1750	0.06	5763.84
28°C, 48 h	Control	11(0)	<1.00	—	—	11(0)	<1.00	—	—	—	—	—
	Low	11(0)	2.17	0.29^g^	0.30	11(1)	2.07	0.36	0.38	0.1391	0.10	30.42
	Medium	11(0)	3.53	0.10	0.10	11(0)	3.47	0.09	0.11	0.0824	0.06	437.23
	High	11(0)	4.67	0.08^g^	0.11	11(0)	4.59	0.10	0.14	0.0966	0.08	7869.00
28°C, 60 h	Control	11(0)	<1.00	—	—	11(0)	<1.00	—	—	—	—	—
	Low	11(0)	2.16	0.29^g^	0.29	11(1)	2.07	0.36	0.38	0.1843	0.09	27.05
	Medium	11(0)	3.53	0.09	0.10	11(0)	3.47	0.09	0.11	0.1095	0.06	437.23
	High	11(0)	4.67	0.08^g^	0.11	11(0)	4.59	0.10	0.14	0.1088	0.08	7869.00

a Samples were analyzed by harmonized FDA-BAM Ch. 18 and ISO 21527 methods using 0.1% peptone water as the sample diluent.

b *n* = Number of laboratories that reported complete results. Outliers are in parentheses.

c Log_10_ yeast and mold cfu/g.

d Significant difference (*P *<* *0.05).

e Results presented as cfu/g.

f Not applicable.

g Results indicate that the candidate method is more repeatable than the reference methods. s_r_ = Repeatability standard deviation; s_R_ = reproducibility standard deviation.

**Table 2014.05B.** Interlaboratory study results of 3M Petrifilm RYM versus FDA-BAM and ISO 21527 methods for raw almonds

		3M Petrifilm RYM method				BAM/ISO 21527 methods^a^						
Matrix	Lot	*n* ^b^	Mean^c^	s_r_	s_R_	*n*	Mean	s_r_	s_R_	*P*-value^d^	Difference of means	Reverse transformed mean difference^e^
Raw almonds												
25°C, 48 h	Control	12(0)	<1.00	—^f^	—	12(0)	<1.00	—	—	—	—	—
	Low	14(0)	1.45	0.17^g^	0.26	14(0)	1.55	0.19	0.34	0.4165	0.10	–7.30
	Medium	14(1)	2.12	0.26	0.39	14(0)	2.21	0.20	0.24	0.3322	0.09	–30.36
	High	14(2)	3.00	0.18	0.49	14(1)	3.08	0.12	0.31	0.2833	0.08	–202.26
25°C, 60 h	Control	12(0)	<1.00	—	—	12(0)	<1.00	—	—	—	—	—
	Low	14(0)	1.53	0.23	0.28	14(0)	1.55	0.19	0.34	0.8391	0.02	–1.60
	Medium	14(0)	2.20	0.21	0.27	14(0)	2.21	0.20	0.24	0.7789	0.01	–3.69
	High	14(2)	3.04	0.18	0.41	14(1)	3.08	0.12	0.31	0.5418	0.04	–105.79
28°C, 48 h	Control	12(0)	<1.00	—	—	12(0)	<1.00	—	—	—	—	—
	Low	14(0)	1.58	0.16^g^	0.21	14(0)	1.55	0.19	0.34	0.7381	0.03	2.54
	Medium	14(0)	2.17	0.17^g^	0.29	14(0)	2.21	0.20	0.24	0.6139	0.04	–11.73
	High	14(2)	3.01	0.17	0.45	14(1)	3.08	0.12	0.31	0.3904	0.07	–178.97
28°C, 60 h	Control	12(0)	<1.00	—	—	12(0)	<1.00	—	—	—	—	—
	Low	14(0)	1.60	0.17^g^	0.20	14(0)	1.55	0.19	0.34	0.5474	0.05	4.33
	Medium	14(0)	2.21	0.17^g^	0.23	14(0)	2.21	0.20	0.24	0.9483	0.00	0.00
	High	14(2)	3.03	0.18	0.42	14(1)	3.08	0.12	0.31	0.4687	0.05	–130.75

a Samples were analyzed by harmonized FDA-BAM Ch. 18 and ISO 21527 methods using 0.1% PW as the sample diluent.

b *n* = Number of laboratories that reported complete results. Outliers are in parentheses.

c Log_10_ yeast and mold cfu/g.

d Significant difference (*P *<* *0.05).

e Results presented as cfu/g.

f Not applicable.

g Results indicate that the candidate method is more repeatable than the reference methods. s_r_ = Repeatability standard deviation; s_R_ = reproducibility standard deviation.

**Table 2014.05C.** Results of aerobic plate count for collaborating laboratories

Lab	Frozen raw ground beef, cfu/g	Raw almonds, cfu/g
1	3.8 × 10^2^	6.0 × 10^1^
2	1.1 × 10^3^	6.0 × 10^2^
3	<10	3.0 × 10^1^
4	Not reported	Not reported
5	2.8 × 10^3^	2.8 × 10^1^
6	8.0 × 10^1^	2.2 × 10^1^
7	9.1 × 10^2^	1.6 × 10^2^
8	Not reported	Not reported
9	9.0 × 10^2^	2.0 × 10^2^
10	1.3 × 10^3^	4.0 × 10^2^
11	>2500	1.0 × 10^1^
12	Not reported	7.0 × 10^1^
13	9.5 × 10^1^	1.0 × 10^1^
14	7.3 × 10^2^	2.3 × 10^2^
15	3.7 × 10^2^	8.0 × 10^1^

**Table 2014.05D.** Appearance of yeast and mold on 3M Petrifilm RYM Plates

Yeast	Mold
Small colonies	Large colonies
Colonies have defined edges	Colonies have diffused edges
Pink/tan or blue/green in color	Blue/green to variable upon prolonged incubation
Colonies appear raised (three-dimensional)	Colonies appear flat
Colonies have a uniform color	Colonies have a dark center with diffused edges

## Discussion

In this study, the 3M Petrifilm RYM Count Plate method was evaluated at 25 ± 1°C and 28 ± 1 °C for 60 to 72 h and compared to AOAC SMPR 2021.009 for Viable Yeast and Mold Count Enumeration in Cannabis and Cannabis Products (13). Naturally contaminated dried cannabis flower samples were tested at low and high levels at 60 and 72 h or low, medium, and high levels at 72 h. The log counts from the 3M Petrifilm RYM Count Plate method were compared with log counts from DRBC agar.

The 90 and 95% confidence intervals indicated there were no significant differences in detection or enumeration between the 3M Petrifilm RYM Count Plate method and the DRBC agar at the medium and high contamination levels at both temperatures, 25 ± 1°C and 28 ± 1°C, at 72 h. The 90% confidence interval was just outside the acceptance criterion for statistical equivalence at the low level at both temperatures, 25 ± 1°C and 28 ± 1°C at 72 h (−0.577, −0.236 and −0.682, −0.322, respectively). A higher standard deviation was observed on the DRBC agar plates (repeatability standard devidation (s_r_) >0.2), which can lead to wider confidence intervals.

In order to investigate the recovery differences between the 3M Petrifilm RYM Count Plate method and DRBC at low levels further, a second study was conducted at low and high levels at both temperatures, 25 ± 1°C and 28 ± 1°C, at 60 and 72 h. In this study, the 90 and 95% confidence intervals of the bias between the two methods fell between −0.5 to 0.5 log_10_ for each concentration indicating equivalence between the 3M Petrifilm RYM Count Plate method and the DRBC agar at the low and high contamination levels at 25 ± 1°C and 28 ± 1°C, at 60 or 72 h per AOAC SMPR 2021.009 recommended acceptance criteria.

During the second study, the laboratory noted that the DRBC reference plates appeared to have breakthrough growth of *Pseudomonas.* These bacterial colonies were initially mistaken as yeast colonies, which inflated the DRBC counts. DRBC reference plates were recounted with the omission of *Pseudomonas*. To confirm that the omitted colonies were bacteria, three visually distinct colonies were sent for genetic sequencing and identified as *P. aeruginosa*, *P. juntendi*, and *P. lactis.* In the first study, it was noted that the amount of contamination at the low level on DRBC (5360 cfu/g) was higher than expected based on the screening data provided by Steadfast (300 cfu/g Steadfast) while the recovery on 3M Petrifilm RYM Plate (325 and 318 cfu/g at 25 ± 1°C and 28 ± 1°C respectively) was aligned with the value provided by Steadfast for the low contamination level. It is possible that breakthrough growth of *Pseudomonas* was mistaken as yeast colonies and could explain the higher than expected level of contamination recovered on DRBC at the low level during the first study. During that study, there was no investigation of the colony types, and thus all colonies from the DRBC plates were included in the counts.

In the strain studies, of the 71 yeast and mold strains that should have been detected by the 3M Petrifilm RYM Count Plate method, all were detected and had appropriate colony morphology, giving a sensitivity of 100%. None of the 32 non-target strains were detected by the 3M Petrifilm RYM Count plate method, giving a specificity of 100%.

## Conclusions

These studies have demonstrated that the 3M Petrifilm RYM Count Plate method is an accurate, specific, and repeatable method that detects and enumerates yeast and mold at 60 to 72 h from dried cannabis flower. It is recommended that the 3M Petrifilm RYM Plate, AOAC OMA **2014.05**, be granted a matrix extension for the detection and enumeration of yeasts and molds in dried cannabis flower.

## Conflict of Interest

3M Company is the method developer. All authors from 3M Company are salaried employees of the company. North Coast Testing Laboratories, Aurum Laboratories, and Q Laboratories were contracted as independent laboratories to conduct the study per AOAC guidelines and received payment from 3M Company.
